# EBSD Investigation of the Microtexture of Weld Metal and Base Metal in Laser Welded Al–Li Alloys

**DOI:** 10.3390/ma11122357

**Published:** 2018-11-23

**Authors:** Li Cui, Zhibo Peng, Xiaokun Yuan, Dingyong He, Li Chen

**Affiliations:** 1 College of Materials Science and Engineering, Beijing University of Technology, Beijing 100124, China; pengzhibo@emails.bjut.edu.cn (Z.P.); yuanxiaokun@bjut.edu.cn (X.Y.); dyhe@bjut.edu.cn (D.H.); 2 AVIC Manufacturing Technology Institute, Beijing 100024, China; ouchenxi@163.com

**Keywords:** Al–Li alloys, laser welding, weld metal, base metal, grain orientation, texture

## Abstract

Autogenous laser welding of 5A90 Al–Li alloy sheets in a butt-joint configuration was carried out in this study. The microstructure characteristics of the weld metal and base metal in the horizontal surface and the transverse section of the welded joints were examined quantitatively using electron back scattered diffraction (EBSD) technique. The results show that the weld metal in the horizontal surface and the transverse section exhibits similar grain structural features including the grain orientations, grain shapes, and grain sizes, whereas distinct differences in the texture intensity and misorientation distributions are observed. However, the base metal in the horizontal surface and the transverse section of the joints reveals the obvious different texture characteristics in terms of the grain orientation, grain morphology, predominate texture ingredients, distribution intensities of textures, and grain boundary misorientation distribution, resulting in the diversity of the microhardness in the base metal and the softening of the weld metal. However, the degree of the drop in the hardness of the weld metal is highly correlated to the microtexture developed in the base metal.

## 1. Introduction

Lithium (Li) addition to aluminum alloys causes substantial reduction in the density accompanied by large increase in elastic modulus, appreciable improvement of specific strength and specific stiffness of the alloys, making Al–Li based alloys strong candidates used in high-performance lightweight aerospace structures [1,2]. These alloys in the welded form could be further lightening the structures with weight savings. Therefore, welding of Al–Li based alloys is a significant challenge to provide both weight superiorities and cost benefits. Until now many studies have been made on conventional arc welding of Al–Li based alloys using a wide variety of welding processes [3,4,5,6,7,8]. Serious mechanical property degradation and high deformation in arc welding of Al–Li based alloys have been reported [1,3,4,5,6,7,8]. The use of laser welding is particularly attractive for Al–Li based alloys due to the tight focus ability and high power density of the laser beam [1,9,10]. Due to the low heat input and the rapid cooling rate resulting from high travel speeds, the laser welded joint was characterized by a fine grained weld and a narrow heat affected zone (HAZ) [9,10] which makes the softening in HAZ negligible to the tensile strength of the joints [9]. Thus, they make the mechanical properties of the joints superior to those of the other arc welding processes with a lower power density [1,3,9,10,11].

5A90 Al–Li alloy provided by Southwest Aluminum Co., Ltd., Chongqing, China, has the advantages of excellent corrosion resistance and weldability [12]. In order to further understand 5A90 Al–Li alloys and expand its usage, laser welding of 5A90 Al–Li thin sheets has been developed to meet the needs of the medium strength applications in the aircraft and aerospace structures, including the vapor and plasmas characteristics, welding parameters, welding defects prevention, microstructures, and mechanical properties of the welded joints [13,14,15,16]. It has been shown that some distinct differences in the microstructures between the base metal and the weld metal [9,14,15,16] was found, and it is still important to further clarify the microstructural characteristics of the weld metal of 5A90 Al–Li alloys.

The microstructures of the materials are relative routinely characterized by the morphology and distribution of constituent phases. However, an elaborate and complete description of microstructure of a crystalline material must also include the knowledge about crystallographic orientation features and textures of the constituent grains [17]. Currently, there are increasing reports concerning the considerable deformation textures in the Al–Li based alloys, and crystallographic textures in the Al–Li alloys are quite significant to the properties via rendering them anisotropic [17,18]. Given that texture often causes anisotropic mechanical properties, its presence in the weld zones of the Al–Li alloys could be quite significant [19]. The information available on weld metal texture of 5A90 Al–Li alloys is, however, relatively scanty.

In general, the most common description of the texture for materials can be given in terms of various graphical plots via the grain orientation image mappings (OIM), pole figures (PF), misorientation angles and orientation distribution function (ODF) [17,20,21]. Although these approaches provide a useful description of the textures, the extracted texture information is insufficient. It is often desirable to determine the volume fractions of different texture components. Moreover, the difference in texture along the different directions (for example, sample directions including the rolling direction (RD), the transverse direction (TD), and the normal direction (ND)), which is quite essential to controlling of the welded joint performance, is still not very clear. Therefore, the major attempts in the current study are as follows: (1) to investigate the orientation bias of the weld metal grains along different sample directions; (2) to compare the orientation bias of the base metal grains along different sample directions. Summarizing the results of the above researches will provide quite beneficial information about the relationship between local orientation bias (of both grains and boundary planes) and local performance parameters, and will have broad meanings to the joining technique of the similar Al–Li alloys.

## 2. Materials and Methods

**Figure 1:**
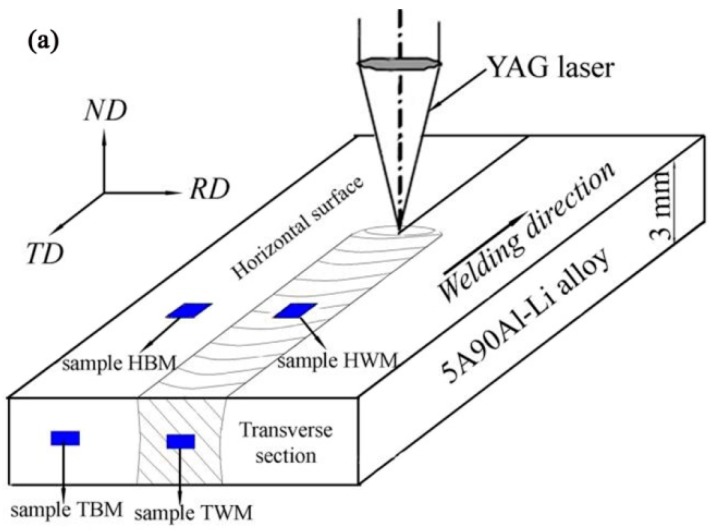

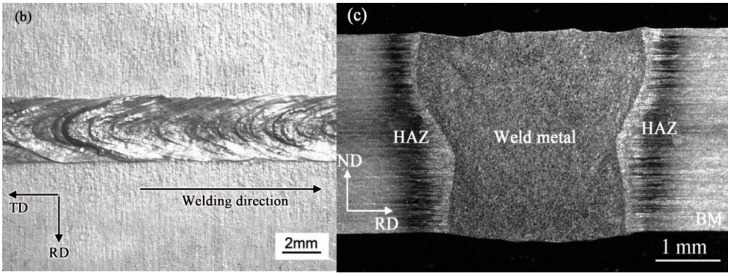
Laser welded 5A90 Al–Li alloys: (**a**) Schematic diagram of the locations of EBSD samples; (**b**) Weld appearance of the joint in the horizontal surface; and (**c**) cross-section of the joint in the transverse section.

5A90 Al–Li alloy sheets of 3.0 mm thickness were used in this study. The chemical composition (wt%) of the 5A90 Al–Li alloy is shown in Table 1. Before welding, the specimens were chemically removed 0.2 mm thickness from each side using 10~20% NaOH solution (Beijing Chemical Works Co., Ltd., Beijing, China), and immersed in 20% nitric acid solution (Beijing Chemical Works Co., Ltd., Beijing, China), prior to polishing to minimize the presence of porosity. Full penetration I-butt joints were made using a Nd:YAG laser (GSI (Shanghai) Co., Ltd., Shanghai, United Kondom), with the nominal maximum laser power of 4.5 kW. The laser fixed to a six-axial welding robot with an emission wave length of 1.07 μm can deliver in continuous wave mode through an output fiber core diameter of 600 μm. A focusing lens with a focal length of 200 mm was used and the beam parameter product (BPP) of the laser beam at the focal point was 25 mm. The laser head was operated 15° leaning to the normal direction of the horizontal surface of the weld joint to prevent the fiber being burned. A 200 mm focal length lens was employed to focus the beam on the specimen surface. The laser power was 1.8 kW and the travel speed was 45 mm/s. An argon shielding gas at a flow rate of 20 L/min was used to shield the welding pool from the atmosphere and the back shielding gas was supplied by ultrahigh purity argon at a flow rate of 15 L/min during welding. Schematic diagram of laser welded joints of 5A90 Al–Li alloys was shown in Figure 1, where the horizontal surface refers to the RD-TD plane and the transversal section refers to the RD-ND plane.

Throughout the experiments, the welding operation was shielded by the trailing and back shielding gas supplied by ultrahigh purity argon at flow rates of 20 L/min and 15 L/min.

After welding, the visual checking of the weld surface and the X-ray inspection of the joints were performed. The weld ripples and weld width are uniform and the weld surface should be no visible porosity, hot cracking. Additionally, the porosity of the welds should be less than grade II. Consistent with the above two respects, the welded joints were considered as acceptable specimens selected for the further microtexture study by means of the EBSD technique. The locations of the EBSD samples selected from the base metal and weld metal of the welded joints are depicted in Figure 1, where the coordinate system consists of three (x,y,z) axes coinciding with the sample directions. As the next step, the samples for EBSD analysis were removed from the base metal and weld metal of both the horizontal surface and the transverse section, and were designated as sample HBM (abbreviated for horizontal base metal), HWM (abbreviated for horizontal weld metal), TBM (transversal base metal) and TWM (transversal weld metal), respectively. Afterwards, the four samples were mechanically polished and subsequently, electropolished by immersion in a 30% nitric acid in methanol (Beijing Chemical Works Co., Ltd., Beijing, China), solution cooled to −25 °C at a voltage of 20 V for 30 s. These treatments yielded sample surfaces suitable for EBSD mapping.

The EBSD analysis was performed by using the high speed detector (EDAX Genesis 2000 system) (EDAX Co., Ltd., Salt Lake, USA), incorporated in a thermal field scanning electron microscope (SEM, JEOLJSM 6500F) (JEOL Co., Ltd., Tokyo, Japan), with an accelerating voltage of 15 kV. To ensure the accuracy of the EBSD measurements, the data were collected with a step size of 1 micron. The EBSD data were then transported into the TSL OIM Analysis 5.3 software (EDAX Co., Ltd., Salt Lake, USA), for further analysis. To describe the grain orientation and texture at different locations, the microstructures of the samples were indicated by the inverse pole figure maps and the image quality maps. For the selected samples, their corresponding orientation and texture were illustrated via the crystal orientation maps showing the spatial distributions and volume fractions for the ideal fcc rolling components. To describe the orientation bias of boundary planes at different locations, the misorientation across the observed boundaries was illustrated by the misorientation angle distribution functions (MDFs). Moreover, the grain boundary plane orientation distribution function (namely GBP-ODF, which describes the orientation bias of boundary planes, developed under the auspices of Carnegie Mellon University [22,23,24]), were used to study the grain boundary character distribution (GBCD) of the special Σ3 boundary.

## 3. Results

### 3.1. Grain Orientation, Grain Shape, and Grain Size

**Figure 2:**
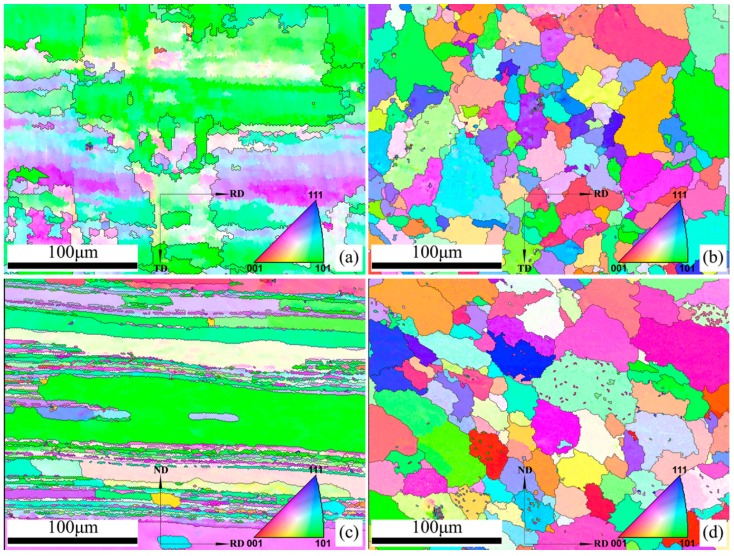
Inverse pole figure maps of base metal and weld metal in 5A90 Al–Li alloys along different sample directions with orientation legend for the cubic symmetry: (**a**) Sample HBM, (**b**) sample HWM, (**c**) sample TBM, and (**d**) sample TWM.

The microstructure of the four samples is depicted by inverse pole figure (IPF) map in Figure 2, where the grain color specifies the orientation according to the coloring indicated in the orientation legend for the cubic symmetry. The IPF maps of the sample HBM and TBM (Figure 2a,c) clearly illustrate that the base metal in the horizontal surface and the transverse section both have strong preferred orientations, however, the base metal has different preferred orientations on these two directions. Sample HBM predominately shows <101> orientations, while sample TBM strongly favors the <112> and <100> orientations. Moreover, the IPF maps of the sample HBM and TBM also reveal the difference in the grain shapes. The microstructure of the sample HBM is characterized by a coarse pancake shape, whereas the grains of the sample TBM present a lamellar structure along the rolling direction. In particular, several long and coarse “deformation bands” are observed in the sample TBM, indicating the microstructure of the base metal in the transverse section is not homogeneous after the rolling deformation. In short, the base metal in the horizontal surface and the transversal section exhibits obvious difference in grain orientation and grain morphology.

For the weld metal, the IPF maps of the sample HWM and sample TWM exhibit similar equiaxed grains (having a wide variety of colors corresponding to varied crystallographic orientations, see Figure 2b,d), indicating that the weld metal in the horizontal surface and the transversal section has nearly the same grains orientation features and grain shapes. This can also be supported by the fact that the grain sizes of the sample HWM and sample TWM are 31.0 μm and 36.8 μm, respectively. It can, therefore, be concluded that the weld metal in the horizontal surface and the transverse section exhibits similar structural features regarding the grain shape, grain orientation, and grain size parameters.

### 3.2. Spatial Distribution of the Textures

**Figure 3:**
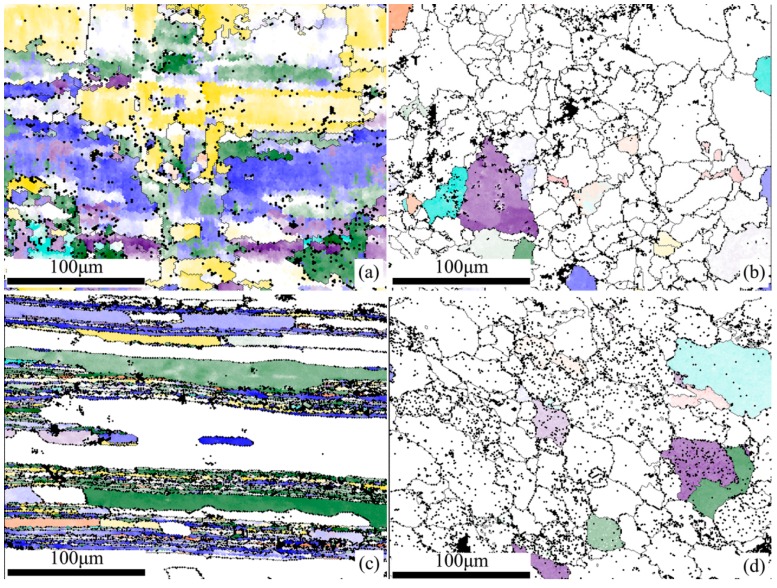
Spatial distributions of typical fcc rolling components of base metal and weld metal in 5A90 Al–Li alloys along different sample directions, with orange for copper, green for S1, purple for S2, blue for S3, cyan for Taylor, yellow for Brass, and red for Goss: (**a**) sample HBM; (**b**) sample HWM; (**c**) sample TBM; and (**d**) sample TWM.

It is generally reported that the rolling texture of fcc symmetries mainly contains the Goss ({011} <100>), brass ({011} <211>), S ({123} <634>), and copper components ({112} <111>). To quantitatively determine the volume fractions of the typical texture components and to analyze the spatial distribution of the rolling textures, the crystal orientation maps of the four samples showing the spatial distribution of fcc rolling components are illustrated in Figure 3 (in which each grain color specifies a texture component), and the corresponding volume fractions of the rolling components are listed in Table 2. Together with the brass, copper, and Goss, the S1 ({241} <112>), S2 ({231} <124>), S3 ({231} <346>) component and Taylor component ({4411} <11118>) are also presented in the base metal and weld metal of the joints. As shown in Figure 3, the colors are orange for copper, green for S1, purple for S2, blue for S3, cyan for Taylor, yellow for brass, and red for Goss.

It can be seen that the sample HBM (Figure 3a) exhibits significant Brass components and the overall intensity of the components is approximately 69.4%. This is obviously a consequence of the higher deformation degree during the rolling processing subjected to 5A90 Al–Li alloys [18]. In sample TBM, the rolling components show weak distribution intensities with a sum percentage about 38.3%, with the S components including S1, S2, and S3 as the predominate ingredients.

For the weld metal, the overall texture intensity of the sample HWM and the sample TWM is 12.3% and 9.4%, exhibiting a relatively weak texture in the weld metal (see Figure 3b,d and Table 1). In general, the overall texture of the weld metal is basically decided by the orientations of the grains [25] in the columnar zone [19] and the weld metal zone having the equiaxed grains is likely to have an almost random texture, while brass, copper and S components are normally encountered in the deformation texture of fcc materials [20,26]. The present work conforms this and the brass, copper, S and Goss components are in presence in the weld metal, which suggests some similar texture components in the weld metal were formed as base metal during the laser welding.

Previous reports [19,27] have suggested that the welded joint could develop several major textures and strong texture might exist at the base metal, HAZ and columnar grain zone of the weld metal, whereas the grain orientation at equiaxed grain zone in the center of weld metal was relatively random. The present work also confirms that the original base metal structure has been eliminated and replaced by a very fine equiaxed grain structure in the weld metal. All these phenomena remind us that during the solidification of aluminum alloys, the equiaxed grains formed in the weld metal are more prone to have random distributions with weaker intensities.

Generally speaking, the equiaxed grains are more likely be generated by the continuous dynamic recrystallization [26]. However, during the solidification of the welding pool, almost all equiaxed grains are those newly-nucleated and grown, hence the grain orientation is random [17]. Therefore, the equiaxed grain cannot be generated by the dynamic recrystallization occurred during laser welding. The formation of equiaxed grains in the weld metal could be ascribed to the heterogeneous nucleation mechanism aided by equilibrium Al_3_Zr phase as well as the growth of pre-existing nuclei created by dendrite fragmentation, or by grain detachment resulted from Nd:YAG laser welding processes [16]. Hence, the random texture developed in the weld metal can be quite different from the normal random texture generated by the continuous dynamic recrystallization.

### 3.3. Texture Fiber Analysis

It is generally known that the rolling texture of fcc symmetries is near the location of so-called α-fiber and β-fiber textures in the Euler space [18,20]. The α-fiber mainly contains the Goss and the Brass orientations, while the β-fiber mainly contains brass, S and copper components.

**Figure 4:**
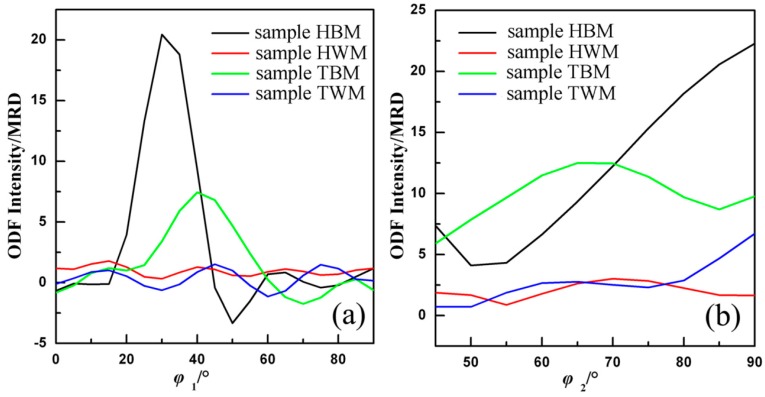
Texture fiber analysis of base metal and weld metal in 5A90 Al–Li alloys along the different sample directions: (**a**) α-fiber; and (**b**) β-fiber.

Figure 4 shows the α-fiber and β-fiber texture analysis of the four samples (in reduced Euler spaces). It can be directly observed that both α-fiber and β-fiber textures are much stronger in base metal (sample HBM and sample TBM) than those of the weld metal (sample HWM and sample TWM). This is not surprising since the higher degree of deformation preferentially developed textures in base metal. Moreover, both α-fiber and β-fiber textures could be modified by different rolling rates, during which the major crystallographic features could be involved. Figure 4 clearly shows the difference in the fiber texture between sample HBM and TBM. On the α-fiber (Figure 4a), the intensity of sample HBM is obviously stronger than that in sample TBM when the *ϕ*_1_ angel ranges from 20° to 40° (meaning that the brass texture is more prevalent), while on the β-fiber (Figure 4b), the intensity of sample TBM is stronger than that of sample HBM when the *ϕ*_2_ angel ranges from 50° to 70° (meaning that the S texture is more prevalent). The outcome is consistent with the results in Table 1, and might indicate that S and Brass textures are preferentially developed with referring to the rolling direction in the base metal.

### 3.4. Orientation Bias of Boundary Planes

**Figure 5:**
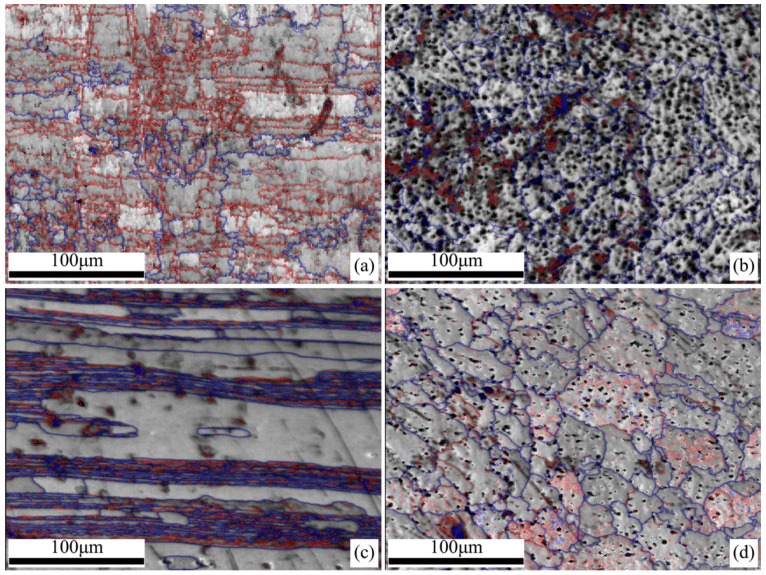
Grain boundary traces imposed on image quality map of base metal and weld metal in 5A90 Al–Li alloys along different sample directions: (**a**) Sample HBM, (**b**) sample HWM, (**c**) sample TBM, and (**d**) sample TWM, with high angle (>15°) grain boundaries shown as blue and low angle (2~15°) boundaries as red.

In the polycrystalline structure, the boundary can be defined either as a high-angle boundary when the misorientation angle across the boundary is higher than 15°, or a low-angle grain boundary when the misorientation angle across the boundary is between 2° and 15°. The image quality maps of the samples are displayed in Figure 5, where the high-angle and low-angle grain boundaries are highlighted in different colors.

It is evident that the sample HBM (Figure 5a) has prevalent low-angle boundaries with a fraction about 67%. As a contrast, the fraction of the low-angle boundaries is merely 30% for the sample TBM with an inhomogeneous lamellar structure (Figure 5c). Furthermore, the microstructure of the sample TBM shows several severely coarse “deformation bands” separated by thin lamellae. The lamellae grains contain high fractions of low-angle boundaries (up to 61%), while little low-angle boundaries and high-angle boundaries within the coarse “deformation bands” can be observed. Thus, a pronounce difference in the grain boundary misorientation of the base metal between the horizontal surface and the transversal section of the welded joint could be found.

For the weld metal, specific difference in the boundary misorientation distributions can be observed between sample HWM (Figure 5b) and sample TWM (Figure 5d). Here, the fraction of the high-angle boundaries for samples HWM and TWM are 67.6% and 53.8%, respectively, that is to say, the weld metal in the horizontal surface has much higher fraction of the high-angle boundaries. A comparison of these results with those of the base metal demonstrates the clear shift in the boundary misorientation distributions from low to high angles. Another difference is, the clustering of the low-angle boundaries is often found within grains, whereas the high-angle boundaries are more prone to occur along grain contours, or, between grain pairs. Hence, there might have a distinct difference in the grain boundary misorientation of the weld metal along different directions.

**Figure 6:**
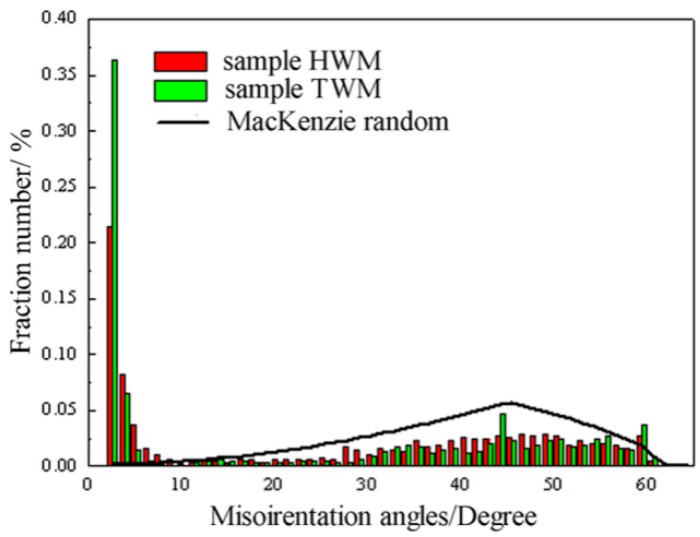
Misorientation angles distributions of the laser weld metal of 5A90 Al–Li alloys along different directions.

A frequency distribution of misorientation angles for sample HWM and sample TWM with a superimposed random McKenzie distribution [28] is shown in Figure 6. The average misorientation angles deduced from the histograms are 30.4°, and 24.9° form sample HWM and sample TWM, respectively. Figure 6 overall illustrates that the misorientation distribution of sample HWM and sample TWM is much different from random distribution. In addition, the relative frequency of intermediate (30° to 50°) for the sample HWM is higher than that of the sample TWM. Meanwhile, the relative frequency of low-angle boundaries for sample HWM is lower than that of the sample TWM. The above results on the whole illustrate the heterogeneity of misorientation of the weld metal on different directions.

**Figure 7:**
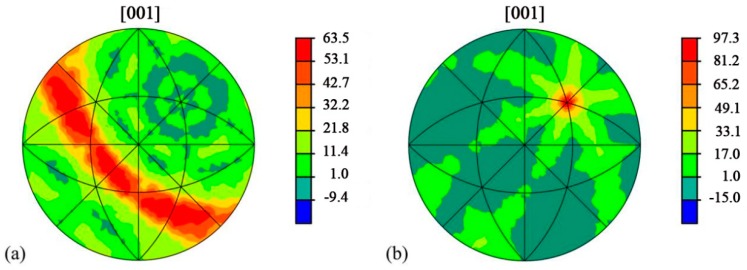
Grain boundary plane orientation distribution function along different sample directions in the base metal of 5A90 Al–Li alloys, showing orientation texture of boundary planes plotted along the [001] direction: (**a**) sample HBM, and (**b**) sample TBM. Texture intensities are given in unit of MRD.

**Figure 8:**
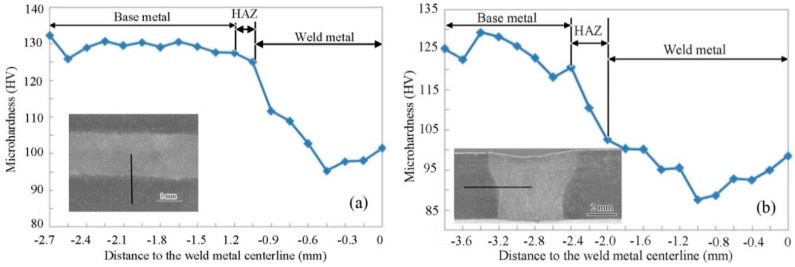
Hardness profiles along different sample directions in the welded 5A90 Al–Li alloys: (**a**) sample HBM, and (**b**) sample TBM.

The GBP-ODF requires large amount of boundary quantity for a complete analysis [22] and in the current work, only sample HBM and sample TBM can meet this standard. The GBP-ODFs of Σ3 boundaries in the sample HBM and sample TBM were calculated and the outcomes are presented in Figure 7. In the sample HBM, the Σ3 boundary shows a tilt boundary feature [29], with distribution intensity in units of 63.5 multiples of a random distribution (MRD); while in the sample TBM, the Σ3 boundary shows a twist boundary character [29], with distribution intensity of 97.3 MRD. Since the Σ3 boundary can be regarded as a 60° rotation along the <111> axis [20], the above results show that the Σ3 boundary exhibit simple geometries. Nevertheless, Σ3 tilt (in sample HBM) and Σ3 twist (in sample TBM) may correspond to specific structures and, consequently, to special physical properties of boundaries. The indexed twist and tilt Σ3 boundaries in the cubic case clearly illustrated that the misorientation distribution in the base metal is not homogeneous along different directions, which could cause the diversity of mechanical properties along different directions in the weld metal. The microhardness across the welded joint from left to right including the base metal, HAZ and weld metal for sample HWM and sample TWM were measured, and the results are shown in Figure 8a,b, respectively. For the base metal, the average hardness value of the base metal is 128 HV in sample HWM, while the average hardness value of the base metal in sample TWM is 117 HV. It indicates that the indexed twist and tilt Σ3 boundaries in the base metal along different directions have resulted in the diversity of the microhardness due to the homogeneous microstructure along different directions. For the weld metal, the microhardness along different directions is affected strongly by its own base metal. As observed in Figure 8, the hardness value measured in the weld metal is ~102 HV for sample HWM, whereas the hardness value measured in the weld metal is ~92 HV for sample TWM. Thus, it can be seen that the hardness value of weld metal is evidently lower than that of the base metal, meaning the softening of the weld metal. Although both sample HWM and sample TWM reveal the softening of the weld metal, the degree of the drop in the hardness of the weld metal is highly correlated to the microtexture developed in the base metal.

## 4. Conclusions

Based on the above results and discussion, the following conclusions can be made from this work:

(1) For the base metal, there is an obvious difference in the grain morphology and orientation in the horizontal surface and the transversal section. However, the weld metal in the horizontal surface and the transverse section exhibits similar structural features regarding the grain shape, grain orientation, and grain size parameters.
(2) For the weld metal, there is an obvious difference in the texture intensity in the horizontal surface and the transversal section, despite the weld metals exhibit the similar grain shapes, grain orientations and grain size. Moreover, the texture intensity are much weaker compared to those of the base metal. Particularly, the brass, copper, S and Goss components observed in the base metal are also presented in the weld metal.
(3) For the boundary plane misorientation, the low-angle boundaries are most predominant in the base metal in the horizontal surface. The large fraction of high-angle boundaries of the weld metal in the horizontal is higher than that of the transversal section of the welded joint. The misorientation distribution of the weld metal is much different from random distribution.
(4) The overall GBP-ODF of Σ3 boundary is not homogeneous in the base metal, resulting in the diversity of the microhardness in the base metal. In addition, the hardness value of weld metal is evidently lower than that of the base metal, meaning the softening of the weld metal. Although both sample HWM and sample TWM reveal the softening of the weld metal, the degree of the drop in the hardness of the weld metal is highly correlated to the microtexture developed in the base metal.

## Figures and Tables

**Table 1:** Chemical composition of 5A90 Al–Li alloys (mass fraction/%).

Mg	Li	Zr	Fe	Si	Cu	Ti	Al
4.9–5.4	1.8–2.2	0.08–0.13	≤0.12	≤0.09	≤0.05	≤0.05	Bal

**Table 2:** Volume fraction of the typical fcc rolling components (%).

Sample	Copper	S1	S2	S3	Taylor	Brass	Goss
HBM	0.3	12.6	4.9	22.0	2.4	27.2	0.0
HWM	1.6	2.4	2.3	2.1	2.0	1.3	0.6
TBM	1.6	13.7	2.5	13.7	0.8	6.0	0.0
TWM	0.4	1.5	3.0	0.8	3.2	0.2	0.3
